# Supplementary material for: Role of MPK4 in pathogen-associated molecular pattern-triggered alternative splicing in Arabidopsis
Source: PLoS Pathog. 2020 Apr 17;16(4):e1008401. doi: 10.1371/journal.ppat.1008401 (PMC7164602; doi:10.1371/journal.ppat.1008401)
Supplement: Attachment — Submitted filename: Responses_to_reviews_151019.MC.doc [file ppat.1008401.s013.doc]

Dear Dr. Hirt:

Thank you very much for submitting your manuscript "Role of MPK4 in pathogen-associated molecular pattern-triggered alternative splicing in Arabidopsis" (#PPATHOGENS-D-19-00086) for review by PLOS Pathogens. As with all papers submitted to the journal, yours was evaluated by a PLOS Pathogens Associate Editor in consultation with the Editorial Board. Your article was also evaluated by independent reviewers.  The reviewers appreciated the attention to an important topic, but they raised substantial concerns about the paper.  Based on the reviews, I regret that we will not be able to accept the manuscript for publication in the journal at this time.

While the reviewers felt the topic is of interest, they raised substantial concerns that would require major new experiments to address and thus are beyond the scope of a revision. Reviewer 1, point 2 and reviewer 2 raise questions about the MAPK4-dependence of flg22-induced DAS events. The strong concern of reviewer 2 about observing induced changes in mpk4 plants with a CPR phenotype has merit and could be addressed, at least partially, by following the suggestions of reviewer 2. Additionally, reviewers 1 and 3 express concerns regarding interpretations about the nature of retained introns and assertions about NMD and the link between DAS genes and the proteome. Lastly, I concur with reviewer 1 that functional analysis to validate the consequences of DAS of select genes would greatly strengthen the solid descriptive groundwork. Given the high interest in the topic, however, we would possibly consider a new submission if it were improved to meet the major criticisms of the
current manuscript.

The new manuscript would receive a new manuscript reference number but will likely be assigned to the previous editors. It will be their task to determine whether or not to send the manuscript to review and, if so, whether or not to include any new reviewers. We cannot promise peer review or further consideration for publication at that time.

The new submission should make reference to the previous manuscript number in the cover letter and provide a detailed response to the previous criticisms included in the cover letter.  

The reviews are copied below, and we hope they may help with any future submissions of this paper to our journal or elsewhere. Please use the link below to see if there are any accompanying reviewer attachments (if no attachments are present, all reviewer comments can be found below).


Reviewer #1: The manuscript „Role of MPK4 in pathogen-associated molecular pattern-triggered alternative splicing in Arabidopsis” by Bazin et al. describes a transcriptome-wide study of alternative splicing changes in response to treatment with the PAMP flg22. The authors report that ~500 genes show changes in the patterns of alternative splicing. Based on the analysis of splicing patterns in mpk mutants, the authors conclude that MPK4 is a key regulator of PAMP-induced alternative splicing.

Research in the past decade has identified alternative splicing as a common post-transcriptional gene control mechanism in plants. Many transcriptome-wide studies of alternative splicing in plants have been reported, revealing that the splicing output is tightly regulated and basically responds to every signal/condition that has been tested. While these studies further highlighted the enormous potential of alternative splicing in gene regulation, only little is known about the regulatory mechanisms underlying alternative splicing changes as well as its functional role. The current study is interesting as it uses a well-studied PAMP as elicitor of alternative splicing. However, the findings described in this manuscript are mainly descriptive and do not provide any novel insight into the mechanisms and the functions of the alternative splicing changes. Furthermore, their experimental design has several limitations (details see below), which brings up the question how reliable
several of their conclusions are. From my point of view, this study only supports the conclusion that flg22 induces numerous changes in alternative splicing, as many other signals and conditions do.

Reviewer #2: (No Response)

Reviewer #3: The paper presents RNA-seq analyses of wild-type Arabidopsis plants treated with flg22 and of mutants of MAPK3, 4 and 6. The former, apart from differentially expressed genes, also identifies over 500 differentially alternatively spliced genes. The major results of the paper are 1) that the mpk4 mutant clearly has a far greater effect on AS than the mpk3 and mpk6 mutants, and 2) the strong overlap between the DAS genes from the mpk4 mutant and the flg22-induced DAS genes shows that MAPK4 is important in regulating splicing/AS in response to PAMPs. The paper goes on to validate some of the AS events and point out some key splicing factor and immunity-related genes such as kinases and transcription factors that are affected by AS. The key results are important and clear.

Major Issues: Key Experiments Required for Acceptance

Reviewer #1: 1) The analysis of the alternative splicing changes is incomplete and would need a better description. The following points should be considered. The authors report 546 differentially alternative spliced (DAS) events in comparison of mock- and flg22-treated wild type plants. However, the authors do not discuss how many alternative splicing events they detect in the different samples. Furthermore, among the regulated events, a very high rate of intron retention (85%) is found. The authors should discuss this in light of the frequency of intron retention for all detected events as well as in the context of other studies. No information is provided how the regulated intron retention events change. Does flg22 induce a higher or lower rate of intron retention, or is there no tendency?

We have now included an analysis based on AS events and not only the DAS genes. We show that similarly to our DAS gene analysis, a common set of AS event overlap between mpk4vsCol and Col_mockvsCol_flg22. As suggested by the reviewer, we have also analyzed the rate of intron retention in each comparison. We found the majority of differential introns are more efficiently spliced in response to flg22. On the opposite, the mpk4 mutation lead to the retention of most introns in both mock and flg22 treated conditions. When comparing retained and released introns, we found flg22 treatment and mpk4 mutation seems to have opposite effect on IR. This is now included in the text.

2) The conclusion that MPK4 is a major regulator of flg22-induced alternative splicing is mainly based on the comparison of the splicing patterns for flg22-treated mpk4 and wild type. However, the experiments for confirmation of the splicing pattern changes (Fig. 4) show that the mutant and wild type already show different alternative splicing patterns under mock conditions for most of the candidates tested. Thus, it should be tested which alternative splicing events are still flg22-responsive in the mutant. To provide the reader a complete data analysis, Venn diagrams for the following comparisons should be presented: 1) DAS events based on comparison of mock and flg22 for each mutant and wild type. 2) DAS events based on comparison of WT and each mutant, both for the mock and flg22 condition. Currently, only some of these data are provided.

We thank the reviewer for this comment and we have now included these comparisons in the new version of the manuscript.

3) Based on a comparison with a previously published dataset (Drechsel et al., 2013), the authors conclude that many of the alternative splicing variants affected by flg22 treatment are “probably be translated into protein.” There are several reasons, why this is a likely an overstatement. For example, the previous study might have missed the corresponding events, and even more importantly, the absence of NMD targeting is no proof for translation.

We agree with the reviewer that the absence of NMD targeting is not proof of protein translation so we have downsized our statement in the text. In addition, we have performed a flg22 treatment on the *upf1upf3* mutant and measured the isoform ratios of 3 DAS genes important for plant immunity (Fig 6CDE). The results show that under our experimental conditions, the alternative isoforms of these transcripts are not targeted by UPF1/UPF3 dependent NMD machinery.

4) The authors checked their list of DAS genes for immunity-related genes. RPP4 and some other candidates were found. However, an independent validation of the RNA-seq data is missing and no functional analysis is provided. What are the consequences of these alternative splicing changes?

We now have performed RT-qPCR validation of the RNA-seq data for three genes involved in plant immunity CRK29, CIPK18, and SERK4 and discuss potential functional consequences of the alternative splicing of them.
5) The analysis of isoform switches has a substantial overlap with the DAS analysis based on the MATS software. However, I’m surprised that for 30% of the events where a switch in the major isoform is seen, no AS changes are detected with the other pipeline. The authors should discuss this finding and further look into this aspect. Which of the two approaches is more reliable? Moreover, the isoform switch should have a clear effect on the expression of the corresponding gene. Further analyses for at least 1-2 candidates should be provided, such as determining protein levels in mock- and flg22-treated plants.

This discrepancy between the two approaches can be explained by the conceptual differences between the two algorithms. rMATS is a count based model only looking at spliced reads to quantify DAS events whereas isoswitchAnalyzeR package is an isoform resolution model based on a differential expression analysis from transcript isoform quantification. Such discrepancies between AS analysis tools have already been observed (Liu et al., Bioinformatics 2014 15:364)

Determining protein levels would require antibodies able to detect both isoforms and conditions to discriminate them. We think that these experiments are out of the scope of this ms.

Reviewer #2: The manuscript by Bazin and coauthors describe a role of MPK4 in the regulation of alternative splicing in flagellin triggered responses.

A major problem with the entire study is that mpk4 mutants exhibit autoimmunity triggered by cytoplasmic immune receptors in the absence of MPK4. Thus, any study in that mutant background makes no sense. The authors cannot conclude anything about MPK4. This is still a major problem in the field. A lot of researchers still study different autoimmune mutants and describe regulatory notes, but at the end of the day - all these mutants are alike. Its almost two decades ago that the guard model became apparent and today its evident that most, if not all, autoimmune mutants, represent mutations in favoured effector targets. Thus, in this reviewers opinion, this work only add to confusion.

We agree with the reviewer that an interpretation of autoimmune mutants must be subject to caution. Therefore, we now have performed RNA-seq on the *summ2* and *summ2mpk4* mutants as *summ2* mutants have been shown to almost completely suppress the strong dwarf and auto-immune phenotype of *mpk4* mutants. We conclude from these data that the cytoplasmic immune receptor SUMM2, which guards the MPK4 pathway (22), plays a major role on regulating the DEGs of MPK4 which are already apparent in mpk4 in the absence of flg22 treatment. However, SUMM2 does play no or only a minor role in the flg22-induced AS events regulated by MPK4(See text, Table 1A and Figure 3). These data show that distinct functions of MPK4 are exerted in the non-activated and flg22-activated MPK4 states and clarifies the events seen in mpk4 knock out mutants. Overall, these results further support that MPK4 directly targets AS regulation in response to flg22.

Reviewer #3: 1) The majority (85%) of AS events identified in the flg22 treatment of wild-type plants were intron retentions. This is significantly higher than the proportion of IR events identified generally when looking at AS in Arabidopsis (Marquez and Chamala) which is about 40%. The authors should comment on this. It is important to remember that although IR is an AS event, it reflects non-splicing or poor efficiency of splicing of an intron. In the context of this paper, where AS of some splicing factors is altered and presumably affects the protein levels of these SFs, it might be expected that splicing efficiency of some introns is reduced. There is clearly not a general splicing defect as many introns are unaffected.

As noted by another reviewer, there is a strong bias on IR events in our dataset suggesting that a potential missplicing effect may occur after flg22 treatment. We have now investigated more deeply this observation by looking at the nature of IR events in each comparison. We found the majority of differential introns are more efficiently spliced in response to flg22. On the opposite, the *mpk4* mutation leads to the retention of most introns in both mock and flg22 treated conditions. When comparing retained and released introns, we found flg22 treatment and *mpk4* mutation seems to have opposite effects on IR. To rule-out a general effect on splicing we have also performed a metagene analysis looking at RNA-seq coverage on different intron sets. This approach showed that there is no global defect on splicing but rather a selective effect on certain IR events.

2) Intron retentions most often introduce stop codons. There is data that suggests that transcripts with intron retentions are detained in the nucleus and are not NMD sensitive because they do not “see” the NMD machinery as they are not exported. The authors need to bear this in mind when interpreting their data on the possible functions of some of the isoforms. For example, overlapping the PAMP-induced DAS genes with the NMD targets from Drechsel et al finds 108 of 506. Then, on p7, there is a statement that therefore “79% of AS transcripts will probably be translated into protein”. This is unlikely to be the case for the above reasons (presence of stop codons in the retained introns; likely nuclear detention). This sentence should be removed and other such references to possible functions of IR transcripts should be treated with greater caution and caveats provided.

This consideration is directly relevant to Fig 6C-E where the domain structures of proteins from different isoforms are being presented. All three examples have IRs which introduce premature termination codons to truncate the ORFs and remove all or parts of domains. It is very likely that these transcripts do not leave the nucleus (and are eventually degraded) (or even maybe some are NMD targets). So it is OK to point out the predicted altered domain structures of proteins encoded by AS isoforms with IRs but it necessary to point out that such truncated proteins may never be made.

We agree with the reviewer that the absence of NMD targeting is not proof of protein translation so we have downsized our statement in the text, as mentioned before.

Some of the IRs may be exitrons which would be of interest – see Marquez et al 2015 Genome Research. These IR transcripts can be found on ribosomes and the introns are in frame. The authors should compare their DAS genes lists with the list of exitron-containing genes which would allow a better discussion of protein isoforms.

For the reviewer’s information, here is a Venn diagram comparing our data with the exitron dataset but only a low overlap is observed. We do not think that this information may add to the ms.

Col (Mock vs flg22)

mpk4flg22 vs mpk4mock

Exitrons containing genes

The authors should also note that because a transcript is not in the Drechsel upf1upf3 dataset does not mean that it is not an NMD target – some of the genes being looked at here may not be expressed or have low expression in the NMD mutants.

We have now performed flg22 treatment on the *upf1upf3* mutant and measured the isoform expression of 3 DAS genes important for plant immunity (Fig 6CDE).

3) Low overlap between DEG and DAS genes.  This is not discussed. It has been observed before (Nikita et al 2017 Plant Journal; Calixto et al 2018 Plant Cell) and could be discussed in terms of transcription and AS cascades.

We have now extended our discussion and cited this work appropriately.

4) Transcript usage and isoform switches. The title of the section “PTI is associated with alternative transcript usage…” and of Fig. 7 “….alternative usage of transcript isoforms” talks about transcript usage but deals with isoform switches. The terminology used is not clear. An isoform switch is generally used to describe where the abundance of usually two isoforms flips under some conditions/cell types etc. Differential transcript usage (DTU) is not necessarily an isoform switch – many of the DAS genes found here will have DTU transcripts but are not isoform switches. This should be clarified. In Fig. 7D, isoform-specific RT-qPCR has measured the abundance of isoforms of specific genes in wild-type and flg22-treated wt plants and splicing ratios have been calculated. I don’t understand why the ratios do not have values between 0 and 1? This should be corrected.

We thank the reviewer for pointing this out. This analysis aimed to identify the most important splicing events induced by flg22. Therefore we have looked for isoform switching events ie a switch in the abundance of two transcript isoforms. We have now corrected the text to clarify this point.

Isoform ratio is not the correct terminology, we have now replaced it by relative isoform abundance and modified the results and methods section to explain this more carefully. To monitor changes in splicing by qRT-PCR, we have calculated the relative abundance of one switching isoform versus an alternative one. This explains why the values are not scaled from 0 to 1. ie. If isoform 1 is 4 times more abundant in condition A, then the value is 4. In condition B isoform 2 is 2 time more abundant and hence the value is 0.5.

5) RNA-seq analysis. The methods say that the Illumina reads were mapped with TopHat and transcript structure constructed with Cufflinks and differential expression calculated using Cufflinks. However the upf1upf3 data has been downloaded and re-analysed with kallisto and AtRTD2. The latter is a much more accurate method.  I presume that the different analysis methods used is historical. However, it is time to stop using TopHat (see tweets from Lior Pachter) and Cufflinks because of the issues with missing and mis-assembled transcripts and the effect this has on transcript quantification and downstream DE and DAS analysis (see AtRTD2 paper).  The RNA-seq experiments are good and the main results are really interesting. Re-analysing the flg22 and mpk4 mutant data would not change the main conclusions but would be more accurate in the detail. The authors should consider re-analysing the data but I also appreciate the time required and the presumably historical analysis of the
different sets of data and so would not insist on it.

We agree, due to historical reasons, the *mpk* mutants analysis was done with software which is currently not the most up-to-date. For this reason we decided to use current methods for new datasets involving *mp4*, *summ2* and *summ2mpk4* mutant analysis in the actual version. Nevertheless, using the newer algorithms for all these analyses would not significantly change the main message of our study. In addition, we were also able to validate our initial analysis using RT-PCR and RT-qPCR.

--------------------

<b>Part III – Minor Issues: Editorial and Data Presentation Modifications</b></br>Please use this section for editorial suggestions as well as relatively minor modifications of existing data that would enhance clarity.<br/><br/>

Reviewer #1: a) The following sentence from the abstract is confusing: “Although many DAS genes are targets of nonsense-mediated degradation (NMD), only 19% are potential NMD targets.” It doesn’t become clear that the first half is a general statement. Moreover, transcripts and not genes are NMD targets.

We have now deleted this sentence

b) The authors refer in the text to their comparison of their DAS dataset with a previous report on NMD targets as Fig. 6 – however, this should be Fig. 7.

Corrected

Reviewer #2: (No Response)

Reviewer #3: The AtRTD2 paper is referred to in both its bioRxiv and NAR version – just use the NAR version.

Corrected

P5 – 100nt reads should be 100bp

This was corrected

Fig. 2 – some of the AS isoforms are very clear but some are not so clear (CRT3, ELK4) – an arrowhead next to the RT-PCR bands would help to highlight them

We have edited figure 2 and 4 accordingly.

Fig. 5 – the GO analysis – it is not clear whether the GO analysis is of the mpk4 DAS genes or the 199 genes that overlap between the mpk4 DAS and flg22 DAS. The arrow comes from the overlap but I believe the GO analysis is of all of the mapk4 DAS genes? Also the text talks of 367 mpk4 DAS genes but the Venn has 366.

The arrow on figure S3A points towards the set of DEG and DAS genes, for which the GO analysis was performed. We have corrected the main text to better explain this analysis.

Fig. 6 and 7 are in the wrong order

We have corrected the figure numbering.

Fig. 6E – I presume the transcripts go from right to left – it would be clearer to switch these to left to right.

We have corrected the figure accordingly.

RNA-seq analysis - I also don’t understand why AtRTD2 and Araport11 would both be used in analysing the upf1upf3 data as AtRTD2 incorporates the Araport11 transcripts?

We apologize for the confusion, our analysis now only uses AtRTD2.
__________________________________________________
In compliance with data protection regulations, you may request that we remove your personal registration details at any time.  (Use the following URL: <https://www.editorialmanager.com/ppathogens/login.asp?a=r>) Please contact the publication office if you have any questions.
